# The Antibacterial and Antioxidant Roles of Buckwheat Honey (BH) in Liquid Preservation of Boar Semen

**DOI:** 10.1155/2021/5573237

**Published:** 2021-06-02

**Authors:** Qun Lan, Yingyu Xie, Jiahua Pan, Qiaohui Chen, Tianfang Xiao, Shaoming Fang

**Affiliations:** College of Animal Science (College of Bee Science), Fujian Agriculture and Forestry University, Fuzhou, China

## Abstract

In the present study, we hypothesized that buckwheat honey (BH) should be regarded as a potential alternative to antibacterial and antioxidant agent in liquid storage of boar semen. To this end, boar semen was firstly studied for *in vitro* dose tolerability to BH by measuring sperm progressive motility. The optimum progressive motility of boar spermatozoa was observed in extender with 0.5% and 0.6% BH addition. Afterward, sperm quality parameters, bacterial profile and composition, total antioxidant (T-AOC), catalase (CAT), superoxide dismutase (SOD), and malondialdehyde (MDA) levels of control, BH supplementation, antibiotics supplementation, and incorporated supplementation were compared during liquid storage period, to further investigate antibacterial and antioxidant properties of BH. The results showed that BH supplementation significantly improved sperm motility, acrosome integrity, plasma membrane integrity, inhibited opportunistic bacterial growth, and altered microbial compositions at the end of preservation. Additionally, T-AOC, SOD, and CAT levels were significantly higher in the BH supplementation group than those in the control and antibiotic supplementation group, whereas MDA level exhibited opposite change pattern. Importantly, BH addition to the extender was able to exert a synergistic effect in combination of antibiotic use. Our findings suggested that the appropriate concentrations (0.5% and 0.6%) of BH were added to the extender could act antibacterial and antioxidant roles in liquid preservation of boar semen.

## 1. Introduction

Artificial insemination (AI) is the most important biotechnological application in swine reproduction which brings about rapid genetic improvement in pigs by spreading the use of males with high productive characteristics [1]. The seminal quality plays a key role in achieving satisfactory pregnancy rates by using AI [2]. Due to collection and processing of ejaculates are not strictly sterile manipulation, and microbial contamination is often observed, which could exert negative effects on the quality of boar semen. For instance, high abundances of bacteria present in porcine sperm lead to decreases in sperm motility and viability, but an increase in acrosome defective percentage [3]. In addition, a high concentration of reactive oxygen species (ROS) in spermatozoa that result in oxidative stress has been linked to toxic metabolites and virulent factors of bacterial species [4].To avoid the depreciation of semen samples and improve the fertilizing capacity of stored spermatozoa, antibiotics and antioxidant agents have been used to prohibit bacterial growth and attenuate oxidative stress in semen, respectively. For example, antibiotic and antioxidant agent such as gentamicin and proanthocyanins are frequently added to the extender for the preservation of boar semen [5, 6]. However, with the expansion of the use of antibiotics in seminal preservation, drug resistance of bacteria has emerged as a new issue [7, 8]. Moreover, potential toxic and side effects of several antioxidant agents on sperm have been reported [9]. Thus, to find alternatives to antimicrobial and antioxidant agents for the preservation of semen of swine have attracted widely scientific attention, especially in the context of the spread of African swine fever threatening the global pig industry. Honey as a natural product mainly comprises different types of sugars, amino acids, flavonoids, and phenolic acids, which provide its excellent antimicrobial and antioxidant activities [10]. Therefore, several studies have evaluated the effectiveness and safety of honey as a replacement to antibiotics and antioxidant agent in extender for the preservation of farm animal semen. Nasreen et al. suggested that extender supplemented with honey was able to improve postthaw progressive motility, plasma membrane integrity, and viability of buffalo spermatozoa, and significantly reduce total aerobic bacterial count [7]. Jerez-Ebensperger et al. indicated that the presence of honey in the extender had an antioxidant effect on cryopreserved ram semen and without detrimental effects on sperm quality [11]. Nonetheless, the effect of honey incorporation into extender on the preservation of boar spermatozoa remains unknown.

In this study, we first introduced buckwheat honey (BH) into the extender, and *in vitro* tolerability of boar sperm to BH was determined. In addition, sperm motility, bacterial concentrations, bacterial compositions, acrosome integrity, plasma membrane integrity, total antioxidant capacity (T-AOC), catalase (CAT), superoxide dismutase (SOD), and micromalondialdehyde (MDA) levels in different extenders with or without BH addition during liquid storage period were investigated. The aim of our study was to evaluate the antibacterial and antioxidant effects of BH on boar sperm liquid preservation at 17°C, which would provide a basic knowledge of application of BH as an alternative to antibiotic and antioxidant agent in the extender.

## 2. Materials and Methods

### 2.1. Buckwheat Honey

Natural pure buckwheat honey (BH) was harvested using the sterilized container in beekeeping cooperative in Inner Mongolia province, China, according to standard quality GH/T 18796-2012. The major physicochemical parameters of BH including total sugar, moisture content, pH, and color were verified according to the national standard method (GB 14963-2011, China) in College of Bee Science of Fujian Agriculture and Forestry University (Table S1).

### 2.2. Animals and Semen Samples

Six Landrace boars were raised in Yongcheng Agriculture Animal Husbandry Technology Co. (Fujian Province, China). The commercial formula feeds and water were offered *ad libitum*. Before semen collection, the penis was disinfected using a sponge soaked with 75% ethanol, and all collection wares were sterilized. Three semen ejaculates were collected from each boar by using gloved hand technique. Gelatinous protein was filtered by double layers of sterile gauze to obtain the sperm-rich fraction of the ejaculate. Only semen samples with volume ≥ 100 ml, concentration ≥ 0.5 billion sperm/mL, and motility ≥ 70% were retained. To minimize the effects of individual differences, a 50 mL qualified semen sample from each boar was pooled for further experimental analysis (two pools). Semen samples were stored at 17°C for transportation. All animal experiments were conducted according to the guidelines for the care and use of experimental animals issued by the Ministry of Agriculture and Rural Affairs of China. The project was approved by Animal Care and Use Committee (ACUC) in Fujian Agriculture and Forestry University.

### 2.3. Experimental Design

#### 2.3.1. Experiment I: Tolerability of Boar Semen to Buckwheat Honey

To evaluate tolerability of boar semen to different concentrations of BH, pooled semen samples were aliquoted and diluted to a final concentration of 45 million sperm/mL in the sodium citrate buffer (1.85 g sodium citrate dissolved in 50 mL distilled water, 17°C) which containing BH (*v*/*v*) at 0, 0.1%, 0.2%, 0.3%, 0.4%, 0.5%, 0.6%, 0.7%, 0.8%, 0.9%, and 1%, the osmolarity of each group was shown in Table S2. After 1 h of incubation at 17°C, sperm progressive motility of each treatment was assessed.

#### 2.3.2. Experiment II: Effects of Different Concentrations of BH and Antibiotics Supplementation to Boar Semen Extender on Semen Quality

Beltsville thawing solution (BTS) was prepared by dissolving (37 g glucose, 1.25 g EDTA-2Na, 6.00 g sodium citrate, 0.75 g potassium chloride, and 1.25 g sodium bicarbonate) in 1 L distilled water. Experimental extenders were prepared as follows: control (BTS), E1 (BTS +0.6 g penicillin and 1 g streptomycin), E2 (BTS+0.25% BH +0.3 g penicillin and 0.5 g streptomycin), E3 (BTS+0.3% BH+0.3 g penicillin and 0.5 g streptomycin), E4 (BTS+0.5% BH), and E5 (BTS+0.6% BH). Pooled semen samples were aliquoted and diluted (45 million sperm/mL) with different experimental extenders. During liquid storage at 17°C within 1-5 days, sperm motility, plasma membrane integrity, acrosomal integrity, bacterial concentration, bacterial compositions, T-AOC, CAT, SOD, and MDA levels were measured.

#### 2.3.3. Sperm Motion Parameter

The percentages of total motility spermatozoa (TM) and progressive motility spermatozoa (PM) were assessed by using a modified protocol according to Bucci et al. [12]. In brief, after incubation at 37°C for 10 min, an aliquot (1.5 *μ*L) of semen sample was injected into a counting chamber. For three replicates of each sample, at least 250 spermatozoa in five separate and random fields were analyzed using a computer-assisted semen analysis (CASA) system (HT CASA II Version 3.0). The setting parameters of CASA were described as follows: temperature was 37°C; minimum contrast was 45; both low and medium velocity average pathway (VAP) cut-off were 30; 60 frames/s; straightness threshold was 30%.

#### 2.3.4. Sperm Plasma Membrane Integrity

The hypo-osmotic swelling test (HOST) method was used to evaluate the membrane integrity of the sperm as previously described by Ramu and Jeyendran [13]. Briefly, 200 *μ*L of semen sample was mixed with 1 mL hypo-osmotic solution (HOST, 13.5 g/L fructose, 7.35 g/L sodium citrate, in 1 L distilled water, 94.02 ± 1.11 mos/kg), and the mixture was incubated at 37°C for 30 min. Then, place a drop (10 *μ*L) of mixture onto a microscopic slide which covered with a coverslip. The count of spermatozoa with swollen tails out of at least 200 sperm in each field was recorded by a phase-contrast microscope at 400× magnification.

#### 2.3.5. Sperm Acrosomal Integrity

Sperm acrosomal membrane integrity was tested by using modified Wright's-Giemsa staining assay [14]. 600 *μ*L Wright's-Giemsa solution was added on a prepared sperm smear and incubated at room temperature for 2 min. 1.8 mL phosphate-buffered saline (PBS) was thoroughly mixed with Wright's-Giemsa and hold for 10 min. After that, the slide was rinsed with running distilled water and naturally air-dried. The stained sperm smear was randomly selected, and at least 200 sperms in five different fields of each slide were measured under a phase-contrast microscope at 1000× magnification.

#### 2.3.6. Bacterial Concentration and Compositions

The bacterial concentrations and compositions were investigated to evaluate the antibacterial effects of BH and antibiotics in diluted boar sperm as previously described by Shaoyong et al. [15]. In brief, the bacterial concentration of each group was measured by using LB agar plates on each experiment day. The semen samples were gradient diluted using PBS and then inoculated on plates. After incubation at 37°C for 48-72 h, the plates with 15-250 colonies were retained for bacterial counting. The bacterial DNA of five days preserved semen samples were extracted by using QIAamp Fast DNA Stool Mini Kit (QIAGEN, Germany) according to manufacturers' instruction. Subsequently, 16S rRNA gene sequencing was performed to determine the bacterial compositions.

#### 2.3.7. T-AOC, SOD, and CAT Activities, MDA Level

The SOD, T-AOC, CAT, and MDA levels of 1, 3, and 5 days preserved semen samples were determined by antioxidant assay kits (Nanjing Jiancheng Bioengineering Institute, Jiangsu, China). The extracts of sperm were prepared using Triton X-100 (1%) by centrifuging at 5000 × g for 10 min. Supernatants were collected and mixed with corresponding reaction buffer from different kits. Subsequently, SOD, T-AOC, CAT, and MDA levels were measured by a spectrophotometer (UNICO UV-2100) at 560, 520, 520, and 532 nm wavelengths, respectively. Every parameter of each group was measured for three times. Due to Raoultella, bacteria are able to produce catalase, and to explore the source of Raoultella in the E3 group, the CAT activity of extender E3 which with or without semen addition was measured.

#### 2.3.8. Statistical Analysis

Data processing and analysis were accomplished by Statistical Package for the Social Sciences software, version 25 (SPSS Inc., Chicago, IL, USA). Shapiro–Wilk test and Levene test were performed to analyze normality of data and homogeneity of variances, respectively. Normalization of data was achieved by log2 transformation. The effects of BH and different experimental extenders on sperm motility, plasma membrane integrity, acrosomal integrity, bacterial concentration, bacterial compositions, T-AOC, CAT, SOD, and MDA levels were detected by one-way ANOVA. Multiple comparisons were performed by using Duncan's method and significance threshold was set at *p* < 0.05.

## 3. Results

### 3.1. Experiment I

#### 3.1.1. Tolerability of Boar Semen to Buckwheat Honey

**Figure 1:**
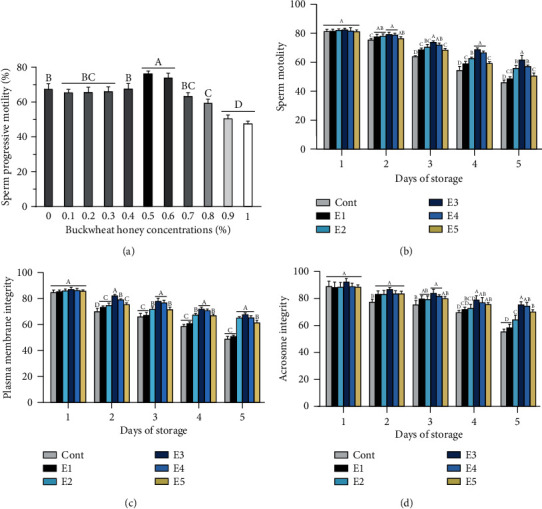
Dose tolerability of boar spermatozoa to different concentrations of BH and alternations in sperm quality parameters during liquid preservation in different extenders. (a) *In vitro* dose tolerability of boar semen to different concentrations of BH in terms of sperm progressive motility (mean ± SD). (b) Sperm motility in different extenders. (c) Plasma membrane integrity in different extenders. (d) Acrosome integrity in different extenders. Bars with different letters differed significantly (*p* < 0.05). Cont: control group; E1 (BTS +0.6 g penicillin and 1 g streptomycin); E2 (BTS+0.25% BH +0.3 g penicillin and 0.5 g streptomycin); E3 (BTS+0.3% BH+0.3 g penicillin and 0.5 g streptomycin); E4 (BTS+0.5% BH); E5 (BTS+0.6% BH).

As shown in Figure 1(a), different concentrations of BH resulted in distinctly changes in sperm progressive motility. Compared to the control group, low (0.1-0.4%) concentrations of BH exhibited no significant differences in sperm progressive motility, but high (0.8-1%) concentrations of BH led to the significant decrease (*p* < 0.05). Interestingly, the highest sperm progressive motility was observed when supplemented with 0.5% and 0.6% of in comparison with the control group.

### 3.2. Experiment II

#### 3.2.1. Effects of Different Concentrations of BH and Antibiotics on Sperm Quality Parameters

Effects of BH and antibiotics supplementation on sperm motility are shown in Figure 1(b). From the beginning of the experiment to the end of the preservation, decline in sperm motility was observed in all groups. All experimental extenders exerted effective roles in ameliorating the progress of decline in sperm motility. Among these, extender E3 which consisted of 0.3% BH, 0.3 g penicillin, and 0.5 g streptomycin showed the minimum extent of decline during the storage period. In addition, sperm motility in extenders uniquely supplemented with BH (E4) was significantly higher than that in extenders uniquely supplemented with antibiotics (E1) at the end of preservation (*p* < 0.05).

The morphological characteristics of sperm after the hypo-osmotic swelling test (HOST) were shown in Figure S1. BH and antibiotic supplementation in the extenders that affect sperm plasma membrane integrity are represented in Figure 1(c). Similar to changes in sperm motility, sperm plasma membrane integrity gradually reduced with prolongation of the storage period. In comparison to the extender with antibiotics supplementation (E1), extenders with BH supplementation (E4 and E5) had better protective effects on sperm plasma membrane integrity. After the 3rd day of storage, comparison of both extenders with BH and antibiotics supplementation, sperm plasma membrane integrity of the E3 group was significantly higher than that of the E2 group at the 4th day of storage (*p* < 0.05).

The morphological characteristics of sperm after boar semen stained with Wright's-Giemsa solution were shown in Figure S2. After 5 days of liquid storage, acrosome integrity in the experimental extenders was better than it in the control extender (*p* < 0.05, Figure 1(d)). Comparison of extenders with antibiotics showed that acrosome integrity was improved (*p* < 0.05) in both E2 and E3 (with 0.25% and 0.3% BH supplementation, respectively) compared with extender E1 (without BH supplementation). When comparing both extenders without antibiotics, acrosome integrity was significantly higher (*p* < 0.05) in E4 (with 0.5% BH) than E5 (with 0.6% BH).

#### 3.2.2. Effects of Different Concentrations of BH and Antibiotics on Sperm Antioxidant Parameters

**Figure 2:**
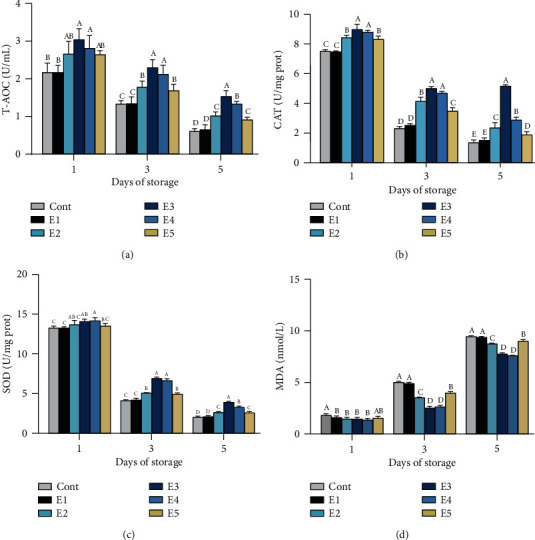
Differences in T-AOC level (a), CAT level (b), SOD level (c), and MDA level (d) among different extenders (mean ± SD). Bars with different letters differed significantly (*p* < 0.05). Cont: control group; E1 (BTS +0.6 g penicillin and 1 g streptomycin); E2 (BTS+0.25% BH +0.3 g penicillin and 0.5 g streptomycin); E3 (BTS+0.3% BH+0.3 g penicillin and 0.5 g streptomycin); E4 (BTS+0.5% BH); E5 (BTS+0.6% BH).

The antioxidant parameters of semen samples including T-AOC, CAT, SOD, and MDA levels were determined on days 1, 3, and 5 of preservation (Figure 2). Interestingly, different from the alterations in sperm quality parameters, the antioxidant parameters differ significantly among all extenders from the 1st day. Although the T-AOC level decline with the preservation time, it maintained higher (*p* < 0.05) in extender E2 (1.02 ± 0.10), E3 (1.53 ± 0.16), E4 (1.33 ± 0.07), and E5 (0.91 ± 0.07) compared with extender control (0.61 ± 0.07) and E1 (0.65 ± 0.13d). The CAT and SOD levels in all extenders showed the similar change pattern with T-AOC, while MDA level that increased with the preservation time and lower values was observed in extender E2 (8.74 ± 0.08), E3 (7.76 ± 0.12), E4 (7.60 ± 0.03), and E5 (8.99 ± 0.16) than extender control (9.45 ± 0.08) and E1 (9.38 ± 0.08) at the end of preservation.

#### 3.2.3. Effects of Different Concentrations of BH and Antibiotics on Bacterial Growth and Composition

**Figure 3:**
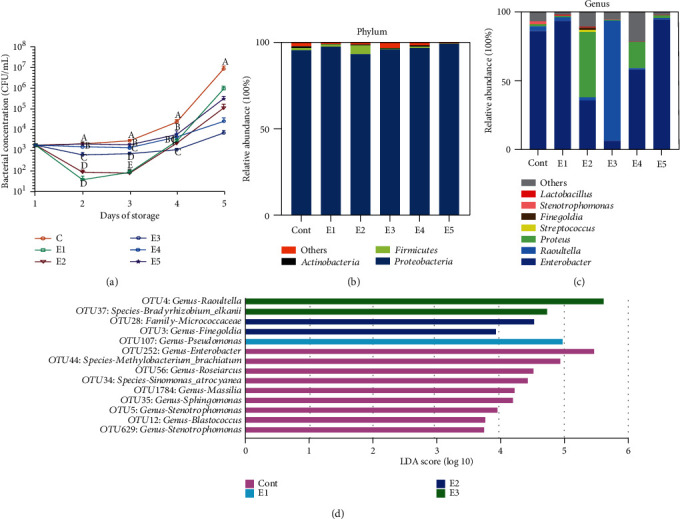
Differences in bacterial concentrations and compositions in different extenders. (a) Bacterial concentration (mean ± SD). (b) Bacterial composition at the phylum level. (c) Bacterial composition at genus level. (d) LEfSe analysis by using relative abundances of OTUs with LDA score > 2.0 (*p* < 0.05). Different superscripts differed significantly (*p* < 0.05). Cont: control group; E1 (BTS +0.6 g penicillin and 1 g streptomycin); E2 (BTS+0.25% BH +0.3 g penicillin and 0.5 g streptomycin); E3 (BTS+0.3% BH+0.3 g penicillin and 0.5 g streptomycin); E4 (BTS+0.5% BH); E5 (BTS+0.6% BH).

As shown in Figure 3(a), bacterial concentration of each group exhibited a decrease in the first two days and an increase in the following three days except the control group. On experimental day 5, the lowest bacterial concentration was observed in extender E3 (8.6 × 10^3^ ± 2.95 × 10^3^ CFU/ML) followed by E4 (2.53 × 10^4^ ± 1.30 × 10^4^ CFU/ML), E2 (1.10 × 10^5^ ± 5.78 × 10^4^ CFU/ML), E5 (3.30 × 10^5^ ± 9.48 × 10^4^ CFU/ML), E1 (7.88 × 10^5^ ± 4.37 × 10^5^ CFU/ML), and control (8.15 × 10^6^ ± 4.09 × 10^6^ CFU/ML).

To further investigate the bacterial composition in the semen samples at the end of preservation, we performed 16S rRNA gene sequencing. At the phylum level, Proteobacteria, Firmicutes, and Actinobacteria were the most dominant phyla in the microbial communities of all preserved semen (Figure 2(b)). Although there no significant changes in relative abundances of these dominant phyla among different extenders (*p* > 0.05, Table S3), the highest relative abundances of Proteobacteria, Firmicutes, and Actinobacteria were presented in extender E5, E2, and E1, respectively. At the genus level, seven predominant genera including Enterobacter, Raoultella, Proteus, Stenotrophomonas, Finegoldia, Streptococcus, and Lactobacillus were detected (Figure 2(c)). Among these, Raoultella, Stenotrophomonas, and Finegoldia showed remarkable alternations in relative abundances in different extenders (*p* < 0.05, Table S4). The highest relative abundances of Raoultella, Finegoldia, and Stenotrophomonas were observed in extender E3, E2, and control, respectively.

Additionally, LEfSe analysis was performed to detect more differential enriched microbial taxa by using relative abundances of OTUs. Fourteen differential enriched OTUs were identified, which consist of nine OTUs enriched in control, one OTU augmented in E1, and two OTUs more abundant in each of E2 and E3, respectively (Figure 2(d)). These OTUs were annotated to different microbial taxa. In the control group, two OTUs were annotated to the genus Stenotrophomonas and one OTU to each of the genus Blastococcus, Sphingomonas, Massilia, Roseiarcus, and Enterobacter. At the species level, one OTU was annotated to Sinomonas atrocyanea and Bradyrhizobium elkanii, respectively. In the E1 group, one OTU was annotated to the genus Pseudomonas. One OTU was annotated to each of the family Micrococcaceae, and the genus Finegoldia was showed in the E2 group, while one OTU was annotated to each of the genus Raoultella, and the species Methylobacterium brachiatum was presented in the E3 group.

## 4. Discussion

Nowadays, antibiotics are routinely used in boar semen liquid storage to inhibit bacterial growth and to improve semen quality. However, the extensive use of antibiotics clearly drives the evolution of antibiotic resistance. Accordingly, it is necessary to identify appropriate substitutes for antibiotics. On the other hand, ROS is generated by sperm during the process of in vitro preservation, which results in oxidative stress in the sperm plasma membrane and functional impairment of the sperm cells. Thus, antioxidant agent supplementation to decrease the impact of different oxidants and protect the sperm cells from oxidative damage is also crucial for the successful liquid preservation of boar semen. In this study, the antibacterial and antioxidant effects of buckwheat honey (BH) were firstly evaluated during boar semen liquid storage to pave the way for using BH as an alternative to antibiotics and antioxidant agents.

It is well known that the suitable levels of supplements for boar semen preservation are very important [16]. Thus, to assess the tolerability of boar semen to BH, different concentrations of BH were added for semen dilution, and progressive motility of sperm was determined. The result showed that boar spermatozoa exhibited good tolerance to 0.5% and 0.6% of BH, but high (0.8-1%) concentrations of BH were deleterious to boar spermatozoa. The high concentrations of BH led to decreases in progressive motility may be ascribed to the blooming of viscosity and hydrogen peroxide of buffer solution with an increase in honey concentrations [17].

Sperm quality is regarded as one of the most important indices to assess the efficiency of semen preservation. To compare BH and antibiotics affect sperm quality, sperm motility, plasma membrane integrity, and acrosome integrity were evaluated during liquid preservation. We found that extender only with BH supplementation had better protective effects on all sperm quality parameters than that only with antibiotics supplementation. Additionally, we also observed the beneficial effects of the incorporation of BH in combination with antibiotics on sperm quality parameter improvement. These findings suggested that BH could maintain sperm quality alone or exert a synergistic effect with the use of antibiotics. Although antibiotics are essential components of semen extenders for the control of bacterial contamination and growth, inflict negative impacts on sperm quality have been reported [18, 19]. Recently, honey is regarded as a potential substitute of antibiotics in the preservation of farm animal semen [7, 20]. The bioactive constituents of BH include sugars, proteins, minerals, and phenolic compounds [21]. Among these, phenolic compounds (e.g., p-hydroxybenzoic acid, benzoic acid, caffeic acid, apigenin, and kaempferol) may play key roles in enhancing cell survival rate and antibacterial activity [22].

Antioxidant characteristic of boar semen intimately related to sperm quality has been noted in previous studies. Pipan et al. found that SOD level was significantly associated with progressive motility and viability during short-term preservation and suggested that SOD in fresh boar semen can be used as a predictor of semen quality after storage [23]. Gloria et al. demonstrated that supplementation of antioxidant (grape marc) in boar diets could reduce the lipid peroxidation of ejaculated spermatozoa and subsequently resulted in improved sperm quality during storage [24]. To uncover the antioxidant effects of BH and antibiotics in semen liquid storage, T-AOC, CAT, SOD, and MDA levels were investigated. BH had stronger antioxidant effects than antibiotics, and the effect could be enhanced by the combination of BH and antibiotic usage. The possible reason for this discrepancy may be attributed to the phenolic and flavone in BH which have strong antioxidant activity. A previous study has indicated that Chinese buckwheat honey contains several flavonoids and phenolic acids, including quercetin, apigenin, kaempferol, myricetin, protocatechuic acid, caffeic acid, p-coumaric acid, and chlorogenic acid, which have been supplemented as exogenous antioxidant substances [25]. Winn and Whitaker suggested that quercetin could improve the motility and IVF rate of postthawed boar semen, while significantly higher SOD and CAT levels of postthawed rooster semen in quercetin supplementation group compared to the control group was observed by Appiah et al. [26, 27]. In another study, apigenin was found to have a beneficial effect on frozen-thawed boar sperm due to its role in modulating oxidative stress [28]. Additionally, Namula et al. demonstrated that when boar semen was preserved at 15°C, the sperm motility, mitochondrial activity, and acrosome integrity were enhanced by chlorogenic acid and caffeic acid supplementation [29].

While performing sperm preservation, the scope is not only to improve sperm antioxidant capacities but also to control bacterial growth simultaneously. Therefore, bacterial concentrations and compositions were compared between BH and antibiotics supplemented extenders to evaluate the antibacterial roles in semen preservation. At the end of preservation, the bacterial concentrations were higher in the antibiotics group than those in the BH group, and the lowest bacterial concentration was observed in the BH and antibiotic incorporated group. BH maintained bacterial concentrations at low level can be attributed to hydrogen peroxide (byproduct of glucose oxidation), bee peptides, lysozyme, phenolic acids, flavonoids, and other nonperoxide bacterial inhibitory components [30, 31]. Flavonoid exerts antibacterial effect as consequences of inhibition of bacterial RNA polymerase and degradation of the bacterial membrane [32]. Similarly, antibacterial activities of phenols and phenolic acids are owing to their important roles in destructing the bacterial cell wall and leading to leakage of cellular contents [33].

With regard to bacterial compositions, we found that the phyla Proteobacteria, Firmicutes, Actinobacteria, genera Raoultella, Stenotrophomonas, and Finegoldia were the most dominant microbial taxa. Although we did not observe any major differences in relative abundances of the predominant phyla, the significantly higher relative abundances of Raoultella, Finegoldia, and Stenotrophomonas were found in extender E3, E2, and control, respectively. The genus Raoultella is a gram-negative bacterium which has been isolated from humans, animals, and natural environments [34, 35]. Unsaturated fatty acids in the plasma membrane were easily oxidized by hydrogen peroxide resulting in sperm membrane rupture and sperm death [36, 37]. Interestingly, Raoultella can produce catalase that may catalyze the decomposition of hydrogen peroxide into water and oxygen [38]. However, the source of Raoultella in E3 group is a puzzle. To explore this problem, the CAT level of extender E3 which with or without semen addition was compared. The result showed that the CAT level of extender E3 with semen addition was significantly higher than that without semen addition (Figure S3, *p* < 0.001). This finding implied that Raoultella derived from boar semen was nourished by BH which may affect sperm antioxidant capacities by catalase secretion. However, further investigations should be performed to verify this speculation.

At the OTU level, 14 OTUs showed significantly different abundances among different extenders. Among these, OTUs annotated to the genus Stenotrophomonas, Blastococcus, Sphingomonas, Massilia, Roseiarcus, and Enterobacter were more abundant in the control group. Most members of these genera are antibiotic-resistant bacteria and detrimental to sperm quality. For example, bacteria from genus Sphingomonas and Stenotrophomonas were frequently detected in bovine semen exhibited a wide range of antimicrobial resistance phenotypes which further impaired sperm motility and suppressed early embryonic development [39, 40]. Species of Enterobacter could degrade antimicrobial agents in swine extenders, damage sperm motility and membrane integrity, and increase sperm agglutination [41, 42]. Taken together, BH supplementation could lead to significant alterations in both bacterial load and bacterial profiles of preserved boar semen.

Our study is limited by small sample size but provides important evidence that incorporation of BH in extender could improve sperm quality, enhance seminal antioxidant capacities, and inhibit the growth of detrimental bacteria. Nevertheless, future studies aiming to elucidate the underlying mechanisms are needed.

## 5. Conclusions

Our results indicated that a suitable concentration (0.5% and 0.6%) of BH as added to extender exert antibacterial and antioxidant roles in boar semen liquid preservation, which highlighted BH should be regarded as a potential substitute for antibiotics and antioxidant agent.

## Data Availability

We submitted 16S rRNA gene sequencing data to the SRA database in NCBI under BioProject accession number PRJNA698400.

## Supplementary Materials

Supplementary Materials The following are available online. Figure S1: morphological characteristics of sperm after the hypo-osmotic swelling test (HOST). (a) No swollen sperm; (b) swollen sperm with curly tails; (c) swollen sperm with an oncotic tail tip. Figure S2: morphological characteristics of sperm after boar semen-stained with Wright's-Giemsa solution; (a) sperm with intact acrosome; (b) sperm with incomplete acrosome. Figure S3: CAT activity of E3 group (adding semen group and nonadding group). Results are expressed as mean ± SD. ^∗∗∗^*p* < 0.001. Table S1: major physicochemical parameters of BH including total sugar, moisture content, pH, and color. Table S2: the osmolarities of sodium citrate buffer with different concentrations of BH addition. Table S3: relative abundances of dominant phyla among different extenders. Table S4: relative abundances of dominant genus among different extenders.
