# Supplementary material for: The Antibacterial and Antioxidant Roles of Buckwheat Honey (BH) in Liquid Preservation of Boar Semen
Source: Biomed Res Int. 2021 Jun 2;2021:5573237. doi: 10.1155/2021/5573237 (PMC8192209; doi:10.1155/2021/5573237)
Supplement: Supplementary Materials — The following are available online. Figure S1: morphological characteristics of sperm after the hypo-osmotic swelling test (HOST). (a) No swollen sperm; (b) swollen sperm with curly tails; (c) swollen sperm with an oncotic tail tip. Figure S2: morphological characteristics of sperm after boar semen-stained with Wright's-Giemsa solution; (a) sperm with intact acrosome; (b) sperm with incomplete acrosome. Figure S3: CAT activity of E3 group (adding semen group and nonadding group). Results are expressed as mean ± SD. ∗∗∗p < 0.001. Table S1: major physicochemical parameters of BH including total sugar, moisture content, pH, and color. Table S2: the osmolarities of sodium citrate buffer with different concentrations of BH addition. Table S3: relative abundances of dominant phyla among different extenders. Table S4: relative abundances of dominant genus among different extenders. [file 5573237.f1.zip › Table S1.docx]

Table S1. Quality parameters of buckwheat honey (BH)

| Parameters | Moiture(%) | Total Sugars(%) | pH | Color |
| --- | --- | --- | --- | --- |
|  |  |  |  |  |
| Normal range | ≤23 | ≥60 | 3.0–6.4 | **——** |
| *Buckwhea*t Honey | 20.3 | 68.25 | 3.98 | Dark brown |
