# Supplementary material for: The Antibacterial and Antioxidant Roles of Buckwheat Honey (BH) in Liquid Preservation of Boar Semen
Source: Biomed Res Int. 2021 Jun 2;2021:5573237. doi: 10.1155/2021/5573237 (PMC8192209; doi:10.1155/2021/5573237)
Supplement: Supplementary Materials — The following are available online. Figure S1: morphological characteristics of sperm after the hypo-osmotic swelling test (HOST). (a) No swollen sperm; (b) swollen sperm with curly tails; (c) swollen sperm with an oncotic tail tip. Figure S2: morphological characteristics of sperm after boar semen-stained with Wright's-Giemsa solution; (a) sperm with intact acrosome; (b) sperm with incomplete acrosome. Figure S3: CAT activity of E3 group (adding semen group and nonadding group). Results are expressed as mean ± SD. ∗∗∗p < 0.001. Table S1: major physicochemical parameters of BH including total sugar, moisture content, pH, and color. Table S2: the osmolarities of sodium citrate buffer with different concentrations of BH addition. Table S3: relative abundances of dominant phyla among different extenders. Table S4: relative abundances of dominant genus among different extenders. [file 5573237.f1.zip › Table S2.docx]

Table S2. The osmolarities of sodium citrate buffer with different concentrations of BH addition （mos/kg）

| 0 | 0.10% | 0.20% | 0.30% | 0.40% | 0.50% | 0.60% | 0.70% | 0.80% | 0.90% | 1% |
| --- | --- | --- | --- | --- | --- | --- | --- | --- | --- | --- |
| 79.75±1.15 | 83.49±1.26 | 92.21±1.67 | 94.99±1.36 | 96.73±1.44 | 106.32±1.56 | 113.19±1.85 | 126.88±1.48 | 136.59±1.28 | 147.44±1.30 | 161.57±1.71 |
