# Supplementary material for: The Antibacterial and Antioxidant Roles of Buckwheat Honey (BH) in Liquid Preservation of Boar Semen
Source: Biomed Res Int. 2021 Jun 2;2021:5573237. doi: 10.1155/2021/5573237 (PMC8192209; doi:10.1155/2021/5573237)
Supplement: Supplementary Materials — The following are available online. Figure S1: morphological characteristics of sperm after the hypo-osmotic swelling test (HOST). (a) No swollen sperm; (b) swollen sperm with curly tails; (c) swollen sperm with an oncotic tail tip. Figure S2: morphological characteristics of sperm after boar semen-stained with Wright's-Giemsa solution; (a) sperm with intact acrosome; (b) sperm with incomplete acrosome. Figure S3: CAT activity of E3 group (adding semen group and nonadding group). Results are expressed as mean ± SD. ∗∗∗p < 0.001. Table S1: major physicochemical parameters of BH including total sugar, moisture content, pH, and color. Table S2: the osmolarities of sodium citrate buffer with different concentrations of BH addition. Table S3: relative abundances of dominant phyla among different extenders. Table S4: relative abundances of dominant genus among different extenders. [file 5573237.f1.zip › Table S3.docx]

Table S3. Relative abundances of dominant phyla in different extenders

| Phylum | C | E1 | E2 | E3 | E4 | E5 | P value |
| --- | --- | --- | --- | --- | --- | --- | --- |
| Proteobacteria | 95.39±2.23 | 97.56±2.46 | 93.23±3.36 | 96.12±1.74 | 96.87±0.68 | 99.18±0.5 | 0.091797 |
| Firmicutes | 1.4±1.07 | 1.36±1.63 | 5.13±3.15 | 0.3±0.07 | 0.84±0.46 | 0.31±0.22 | 0.075274 |
| Crenarchaeota | 0.08±0.09 | 0.04±0.04 | 0±0.01 | 0.76±1.3 | 0.02±0.03 | 0.15±0.25 | 0.601061 |
| Actinobacteria | 0.99±0.34 | 0.27±0.37 | 0.53±0.61 | 0.5±0.24 | 0.69±0.03 | 0.06±0.07 | 0.065736 |
| Acidobacteriota | 0.48±0.23 | 0.02±0.02 | 0.02±0.01 | 0.43±0.19 | 0.29±0.04 | 0 | 0.016587 |
| unidentified Bacteria | 0.25±0.12 | 0.02±0.01 | 0.02±0.02 | 0.42±0.17 | 0.15±0.06 | 0 | 0.008655 |
| Bacteroidota | 0.23±0.24 | 0.27±0.23 | 0.18±0.21 | 0.05±0.04 | 0.14±0.18 | 0.08±0.09 | 0.332658 |
| Campilobacterota | 0.04±0.01 | 0.12±0.16 | 0.13±0.14 | 0.03±0.02 | 0.01±0.01 | 0.02±0.01 | 0.170742 |
| Chloroflexi | 0.07±0.04 | 0 | 0.01±0.01 | 0.24±0.08 | 0.05±0.01 | 0 | 0.007652 |
| WPS-2 | 0.07±0.03 | 0 | 0 | 0.12±0.07 | 0.05±0.01 | 0 | 0.013192 |
| Others | 1.01±0.38 | 0.33±0.11 | 0.76±0.76 | 1.03±0.21 | 0.87±0.1 | 0.2±0.15 | 0.07311 |
