# Supplementary material for: The Antibacterial and Antioxidant Roles of Buckwheat Honey (BH) in Liquid Preservation of Boar Semen
Source: Biomed Res Int. 2021 Jun 2;2021:5573237. doi: 10.1155/2021/5573237 (PMC8192209; doi:10.1155/2021/5573237)
Supplement: Supplementary Materials — The following are available online. Figure S1: morphological characteristics of sperm after the hypo-osmotic swelling test (HOST). (a) No swollen sperm; (b) swollen sperm with curly tails; (c) swollen sperm with an oncotic tail tip. Figure S2: morphological characteristics of sperm after boar semen-stained with Wright's-Giemsa solution; (a) sperm with intact acrosome; (b) sperm with incomplete acrosome. Figure S3: CAT activity of E3 group (adding semen group and nonadding group). Results are expressed as mean ± SD. ∗∗∗p < 0.001. Table S1: major physicochemical parameters of BH including total sugar, moisture content, pH, and color. Table S2: the osmolarities of sodium citrate buffer with different concentrations of BH addition. Table S3: relative abundances of dominant phyla among different extenders. Table S4: relative abundances of dominant genus among different extenders. [file 5573237.f1.zip › Table S4.docx]

Table S4. Relative abundances of dominant genera in different extenders

| Genus | C | E1 | E2 | E3 | E4 | E5 | P value |
| --- | --- | --- | --- | --- | --- | --- | --- |
| Enterobacter | 86.1±6.24 | 93.43±3.99 | 35.62±51.21 | 6.29±2.79 | 58.14±42.95 | 94.41±3.76 | 0.067511 |
| Raoultella | 3.37±0.97 | 2.71±0.92 | 2.28±0.69 | 87.36±5 | 0.89±0.46 | 1±1.6 | 0.027404 |
| Proteus | 1.35±0.91 | 0.86±0.37 | 47.42±40.74 | 0.48±0.11 | 19.04±23.34 | 2.01±2.56 | 0.204631 |
| Streptococcus | 0.04±0.06 | 0.04±0.03 | 1.53±1.75 | 0 | 0.01±0.02 | 0.01±0.02 | 0.079723 |
| Finegoldia | 0.29±0.31 | 0.03±0.03 | 1.67±1.41 | 0 | 0.16±0.13 | 0.03±0.04 | 0.012099 |
| Stenotrophomonas | 1.87±1.16 | 0.1±0.05 | 0.2±0.12 | 0.21±0.09 | 0.17±0.1 | 0 | 0.020235 |
| Candidatus_Nitrosopumilus | 0.08±0.09 | 0.04±0.04 | 0 | 0.76±1.3 | 0.02±0.03 | 0.15±0.24 | 0.601061 |
| Lactobacillus | 0.01±0.01 | 0.71±1.19 | 0.51±0.86 | 0.11±0.17 | 0 | 0.15±0.25 | 0.129501 |
| Escherichia-Shigella | 0.03±0.01 | 0.02±0.01 | 0.25±0.22 | 0.01±0.01 | 0.57±0.47 | 0.01±0.01 | 0.066375 |
| unidentified_UBA10353_marine_group | 0.08±0.08 | 0.04±0.04 | 0.03±0.01 | 0.38±0.56 | 0.02±0.01 | 0.08±0.13 | 0.827817 |
| Others | 6.78±3.24 | 2.01±1.63 | 10.49±7.95 | 4.4±1.59 | 20.98±20.42 | 2.16±2.01 | 0.142911 |
